# Analysis of Arbovirus Isolates from Australia Identifies Novel Bunyaviruses Including a Mapputta Group Virus from Western Australia That Links Gan Gan and Maprik Viruses

**DOI:** 10.1371/journal.pone.0164868

**Published:** 2016-10-20

**Authors:** Thomas Briese, David T. Williams, Vishal Kapoor, Sinead M. Diviney, Andrea Certoma, Jianning Wang, Cheryl A. Johansen, Rashmi Chowdhary, John S. Mackenzie, W. Ian Lipkin

**Affiliations:** 1 Center for Infection and Immunity, Mailman School of Public Health, Columbia University, New York, New York, United States of America; 2 Department of Epidemiology, Mailman School of Public Health, Columbia University, New York, New York, United States of America; 3 CSIRO, Australian Animal Health Laboratory, Geelong, Victoria, Australia; 4 School of Biomedical Sciences, Curtin University, Perth, Western Australia, Australia; 5 The Arbovirus Surveillance and Research Laboratory, University of Western Australia, Nedlands, Western Australia, Australia; 6 Faculty of Health Sciences, Curtin University, Perth, Western Australia, Australia; National Institutes of Health, UNITED STATES

## Abstract

The Mapputta group comprises antigenically related viruses indigenous to Australia and Papua New Guinea that are included in the family *Bunyaviridae* but not currently assigned to a specific genus. We determined and analyzed the genome sequences of five Australian viruses isolated from mosquitoes collected during routine arbovirus surveillance in Western Australia (K10441, SW27571, K13190, and K42904) and New South Wales (12005). Based on matching sequences of all three genome segments to prototype MRM3630 of Trubanaman virus (TRUV), NB6057 of Gan Gan virus (GGV), and MK7532 of Maprik virus (MPKV), isolates K13190 and SW27571 were identified as TRUV, 12005 as GGV, and K42904 as a Mapputta group virus from Western Australia linking GGV and MPKV. The results confirmed serum neutralization data that had linked SW27571 to TRUV. The fifth virus, K10441 from Willare, was most closely related to Batai orthobunyavirus, presumably representing an Australian variant of the virus. Phylogenetic analysis also confirmed the close relationship of our TRUV and GGV isolates to two other recently described Australian viruses, Murrumbidgee virus and Salt Ash virus, respectively. Our findings indicate that TRUV has a wide circulation throughout the Australian continent, demonstrating for the first time its presence in Western Australia. Similarly, the presence of a virus related to GGV, which had been linked to human disease and previously known only from the Australian southeast, was demonstrated in Western Australia. Finally, a Batai virus isolate was identified in Western Australia. The expanding availability of genomic sequence for novel Australian bunyavirus variants supports the identification of suitably conserved or diverse primer-binding target regions to establish group-wide as well as virus-specific nucleic acid tests in support of specific diagnostic and surveillance efforts throughout Australasia.

## Introduction

In 1960, Mapputta virus (MAPV; isolate MRM186), the first of four Australasian bunyavirus-like viruses of the Mapputta serogroup was discovered in *Anopheles (Cellia) meraukensis* mosquitoes collected at Mitchell River Mission (now Kowanyama) in northern Queensland [1, 2]. Subsequently, Trubanaman virus (TRUV; isolate MRM3630) obtained from *Anopheles (Cellia) annulipes* mosquitoes collected in1965 at Kowanyama, and Maprik virus (MPKV; isolate MK7532) obtained in 1966 from *Verrallina* (*Verallina*) *funerea* mosquitoes collected at Maprik, New Guinea, were recognized ([3], https://wwwn.cdc.gov/Arbocat/Default.aspx). The fourth virus was Gan Gan virus (GGV; isolate NB6057), isolated first from *Aedes* (*Ochlerotatus*) *vigilax* mosquitoes collected at Nelson Bay, Port Stephens Peninsula, New South Wales in 1970 [4, 5]. GGV was shown to be responsible for cases of human polyarthritis that tested negative for Ross River virus during the 1983/84 polyarthritis outbreak [6, 7]. All four viruses are assigned to the family *Bunyaviridae*, combined in the “Mapputta group” that is not currently assigned to a genus of the family [8, 9]. However, recent sequence analyses of MAPV MRM186 and MPKV MK7532 indicated a relationship of Mapputta group viruses to the genus *Orthobunyavirus* of the *Bunyaviridae* [10]. Subsequently, TRUV MRM3630 was shown to fall into the same clade, suggesting that two recently characterized viruses from Australia, Buffalo Creek (BUCV; [10]) and Murrumbidge (MURBV; [11]), are also isolates of TRUV [12, 13]. Furthermore, genome sequence analysis of GGV NB6057 indicated that another previously reported virus, Salt Ash (SAHV; [11]), also represents an isolate of GGV instead of a distinct virus [13, 14].

Bunyaviruses are enveloped viruses with a tripartite, negative sense, single-stranded RNA genome. The large (L-)segment of the genome encodes the viral RNA-dependent RNA polymerase (RdRp), the medium (M-)segment encodes two surface glycoproteins (Gn and Gc), and the small (S-)segment encodes the viral nucleoprotein (N). The M-segment of orthobunyaviruses also encodes the non-structural protein NSm, which is thought to be involved in virus assembly [15]. For some orthobunyaviruses, a second non-structural protein is encoded by the S-segment (NSs), which has been shown to modulate the host innate immune response by acting as an interferon induction antagonist [16, 17].

We characterized Australian virus isolates obtained during routine annual mosquito surveillance in Western Australia and New South Wales through sequence analysis. Phylogenetic analyses identified two viruses as TRUV, one as GGV, a fourth as related to GGV and MPKV, and a fifth virus was shown to be closely related to Batai virus that had not previously been demonstrated in Australia.

## Results

Sequence analysis indicated a classical orthobunyaviral genome organization for the studied isolates (SW27571, K13190, 12005, K42904 and K10441), coding the N protein by the S-segment, the Gn-NSm-Gc glycoprotein precursor polyprotein by the M-segment, and the viral RdRp by the L-segment. The S-segment of K10441 also coded for a NSs protein, whereas the other four isolates did not possess an analogous open reading frame (ORF).

In comparison to other orthobunyaviruses as well as among them, the S-segment sequences of SW27571, K13190, 12005 and K42904 showed significant variation in commonly conserved N motifs around invariant amino acids (aa) T_91_/R_94_ and G_150_/P_162_ (aa numbering according to MAPV GenBank no. KJ481921), and in a motif previously proposed to be involved in N multimerization (Y_18_DPNA in MAPV; [18]). Mutation of the widely conserved E_128_ to L (aa numbering according to Bunyamwera virus (BUNV) GenBank no. D00353) was analogous to the E/A_128_ mutation observed in Wyeomyia group viruses [19], which in the orthobunyavirus type species BUNV was found to be associated with a small plaque/high-titer phenotype [20]. Motifs specific to Mapputta group viruses were identified at aa Q_118/119_AE/A/DV/IWRG/E, and K_171/172_QDPEQ.

**Fig 1:**
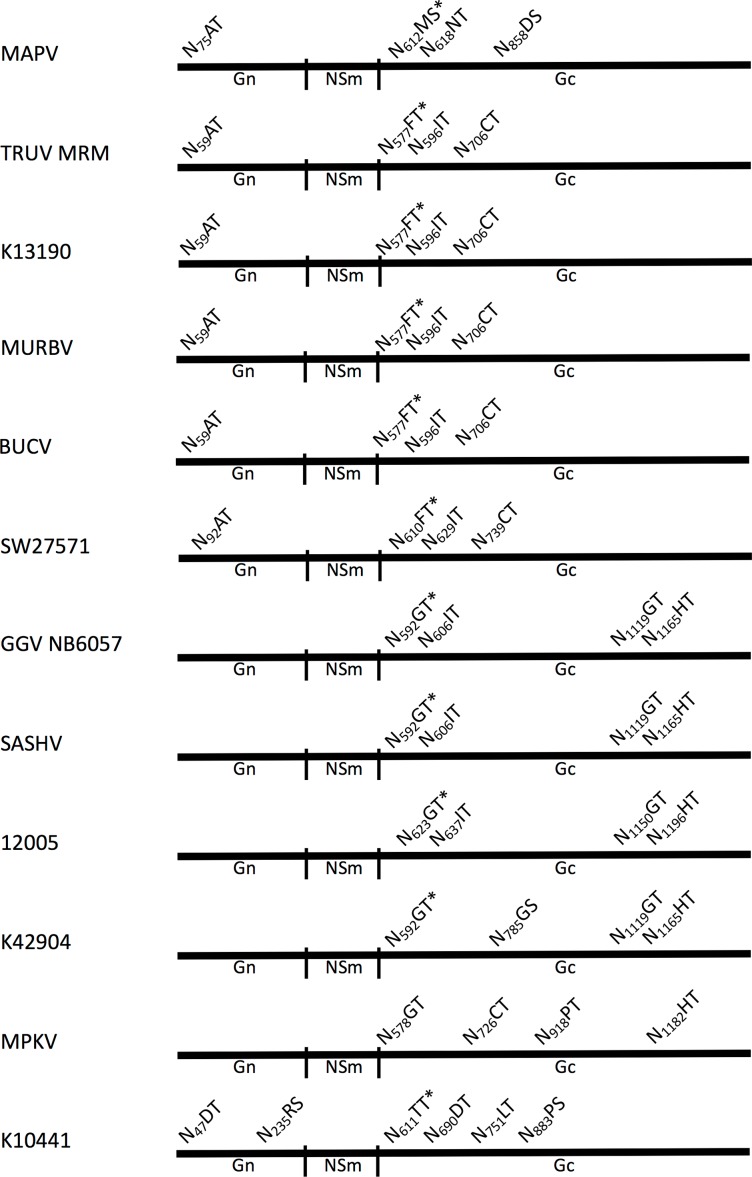
Glycosylation sites of Australian orthobunyavirus M-segment polyprotein sequences. Predicted N-glycosylation sites are indicated along the M-segment sequence by their recognition sequence and aa position. MAPV, Mapputta virus; TRUV, Trubanaman virus; BUCV, Buffalo Creek virus; MURBV, Murrumbidgee virus; GGV, Gan Gan virus; SASHV, Salt Ash virus; MPKV, Maprik virus.

The organization of the M-segment was equivalent to other orthobunyaviruses with signal peptidase cleavage motifs located at the beginning of Gn, NSm, and Gc coding sequences (**Table 1**). Only limited conservation of a site analogous to the protease cleavage site K_293_SLRAAR determined in the California serogroup virus snowshoe hare [21] was observed in all isolates except for K10441 (S_285_LRVAR]. N-glycosylation sites were variable. SW27571 and K13190 were predicted to have a glycosylated Gn, while the Gn of 12005 and K42904 did not show a potential N-glycosylation site (**Fig 1**). The RdRp sequence encoded by the L-segment was characterized by conserved block III domains pre-A, A, B, C, D and E [22, 23], and conservation of the N-terminal endonuclease domain [24].

**Table 1:** Predicted signalase cleavage sites.

	Gn	NSm	Gc
SW27571	SYA/I_16_P	ITA/E_331_C	SFA/I_464_A
K13190	SYA/I_16_P	ITA/E_331_C	SFA/I_464_A
12005	SSQ/A_16_P	INA/D_333_C	VNA/E_469_V
K42904	TTQ/A_16_P	INA/E_333_C	VNA/E_469_V
K10441	n.a.*	IEG/T_331_L	IVA/A_476_T

* n.a., not available

Isolate SW27571 (**Table 2**), obtained from *An*. *annulipes* s.l. mosquitoes collected in 1993 at Thomsons Lake in the City of Cockburn (greater Perth metropolitan area), Western Australia, and isolate K13190 from *An*. *annulipes* s.l. mosquitoes collected in 1993 at Kununurra, Western Australia, were 96%, 96% and 96% identical for their S-, M- and L-segment nt coding sequences, respectively (99%, 98% and 99% aa sequence identity; **S1 Table**), which identifies both as isolates of the same virus. Since SW27571 had been shown to cross-react with TRUV polyclonal antibodies in neutralization assay [25], we also obtained sequences for the original TRUV prototype MRM3630 for which at the time of analysis no sequence was available for comparison. Nucleotide identity between these and recently published TRUV prototype sequences [12, 14] was 100% for all genomic segments. The respective identity for S-, M- and L-segment nt sequences was 98%, 98% and 98% with SW27571 (99%, 99% and 99% aa sequence identity), and 97%, 96% and 96% with K13190 (98%, 99% and 99% aa sequence identity; **S1 Table**). Thus, SW27571 and K13190 represent isolates of TRUV from Western Australia.

**Table 2:** Characterized Australian orthobunyaviruses.

Virus	Isolate	Species of origin	Year	Location	Sequence	Reference
Mapputta (MAPV)	MRM186	*An*. *meraukensis*	1960	Kowanyama, Queensland (QLD)	KJ481921, KJ481922, KJ481923, KP792694, KP792695, KP792696	[10], [12]
Trubanaman (TRUV)	MRM3630	*An*. *annulipes* s.l.	1965	Kowanyama, QLD	KP792682, KP792683, KP792684, KR013237, KR013236, KR013235	[12], [14], this paper
	SW27571	*An*. *annulipes* s.l.	1993	City of Cockburn, Western Australia (WA)	*	[25], this paper
	K13190	*An*. *annulipes* s.l.	1993	Kununurra, WA	*	this paper
	Murrumbidgee (MURBV) 934	*An*. *annulipes* s.l.	1997	Griffith, New South Wales (NSW)	KF234255, KF234254, KF234253	[11]
	Buffalo Creek (BUCV) DPP0186	*An*. *meraukensis*	1982	Darwin, Northern Territory (NT)	KJ481927, KJ481928, KJ481929	[10]
Maprik (MPKV)	MK7532	*Ve*. *funerea*	1966	Maprik, Papua New Guinea	KJ481924, KJ481925, KJ481926	[10]
Gan Gan (GGV)	NB6057	*Ae*. *vigilax*	1970	Nelson Bay, Port Stephens Peninsula, NSW	KR013234, KR013233, KR013232	[14], this paper
	Salt Ash (SASHV) 931	*Ae*. *vigilax*	1992	Salt Ash, Port Stephens Peninsula, NSW	KF234258, KF234257, KF234256	[11]
	12005	*Ae*. *vigilax*	1992	Salt Ash, Port Stephens Peninsula, NSW	*	this paper
	K42904	*Ae*. *vigilax*	2000	Derby, WA	*	this paper
Batai (BATV)	K10441	*Cx*. *annulirostris*	1993	Willare, WA	*	this paper

* Sequence data generated in this study are available through the following GenBank accession numbers: TRUV MRM3630 KU661976, KU661988, and KU661983; TRUV SW27571 KU661981, KU661993, and KU661982; TRUV K13190 KU661977, KU661990, and KU661986; GGV NB6057 KU661978, KU661992, and KU661987; GGV 12005 KU661979, KU661989, and KU661985; GGV K42904 KU640378, KX698605, and KX698606; BATV K10441 KU661980, KU661991, and KU661984.

Isolate 12005 (**Table 2**), obtained from *Ae*. *vigilax* mosquitoes collected in 1992 at Salt Ash, Port Stephens, New South Wales, and isolate K42904, obtained from *Ae*. *vigilax* mosquitoes collected in 2000 at Derby, Western Australia, showed only limited sequence identity to other sequences at the time of analysis. However, determining partial S-, M- and L-segment sequences for GGV prototype NB6057 and comparison to a recently published NB6057 prototype sequence [14] indicated relevant relationships. While sequences of 12005 were 99.4%, 99.3% and 99.6% identical to GGV NB6057 (99.6%, 99.6% and 99.9% aa sequence identity), those of K42904 showed a more distant relationship of 82%, 77% and 79% identity (95%, 88% and 90% aa sequence identity; **S1 Table**). Despite the distance to other GGV isolates, K42904 M-segment sequence was more closely related to GGVs than to any other sequence. K42904 S-, and L-segment nt sequences were equally close to GGVs and MPKV, while the aa sequences showed a closer relationship to GGVs than to MPKV.

Isolate K10441 (**Table 2**), obtained from *Cx*. *annulirostris* mosquitoes trapped in 1993 at Willare, Western Australia showed highest sequence identity (100% nt and 100% aa identity) with a single Australian S-segment sequence entry submitted to GenBank in 2000 (Acc. No. AF325122) and annotated as ‘Bunyamwera virus’. However, all three S-, M- and L-segment coding sequences of K10441 also matched closely with those of characterized Batai virus (BATV) isolates (89–91%, 72–73% and 81% nt sequence; and 97–98%, 79–80% and 93–94% aa sequence identity, respectively; **S1 Table**). Our data suggest that K10441 (and possibly AF325122) represents an Australian variant of Batai virus.

**Fig 2:**
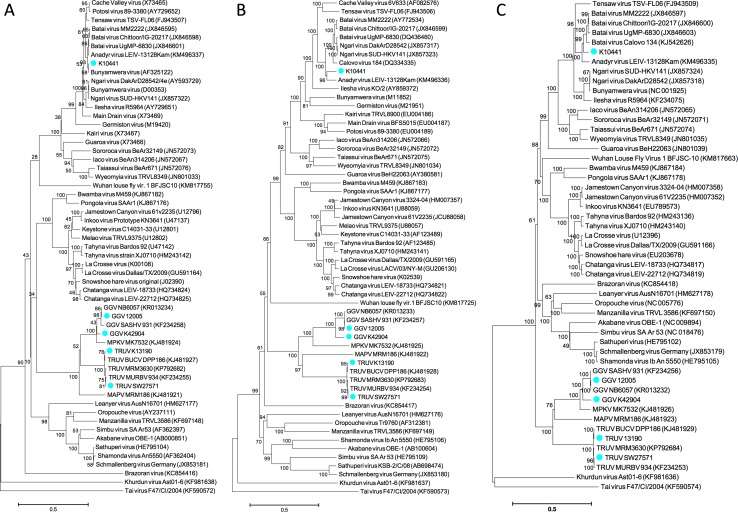
Phylogenetic relationship of Australian Mapputta group and K10441 isolates to other selected orthobunyaviruses. Deduced amino acid sequences of the S- (N ORF; panel **A**), M- (Gn, NSm,Gc polyprotein ORF; panel **B**), and L-segment (RdRp-ORF; panel **C**) were aligned and trees reconstructed with the Neighbor-Joining method applying a Poisson substitution model as implemented in MEGA 6. Bootstrap values resulting from 1000 pseudoreplicates are indicated at the respective nodes; scale bars indicate the number of substitutions per site, and GenBank accession number and isolate name (where known) are given next to the virus name. MAPV, Mapputta virus; MPKV, Maprik virus; TRUV, Trubanaman virus; GGV, Gan Gan virus.

Phylogenetic analyses supported the relationships indicated by pairwise sequence identity analysis. K10441 clustered most closely with BATVs, and SW27671 and K13190 with TRUVs (**Fig 2 and S1 Fig**). While MAPV clearly separates from the other viruses of the group, a separation of MPKV from GGVs was less clear due to the intermediate branching of K42904, at least in the case of S- and L-segment sequence.

## Discussion

Except for K10441, our viruses mapped consistently for all genome segments in a monophyletic clade together with recently sequenced Mapputta group viruses. The sequences of TRUV SW27571 and K13190 were similarly close to TRUV MRM3630 as those of MURBV [12], supporting a classification of all four viruses as isolates of TRUV. Likewise, based on similarity to GGV NB6057 and SASHV [14], 12005 was identified as another isolate of GGV. K42904 was different; though clearly related to GGVs, primarily through M-segment sequence, its S-, and L-segment sequences were also close to those of MPKV. Segment termini or characteristic protein motifs of N or L were not distinctive. Potential M-segment glycosylation sites matched more closely those of GGV except for the lack of the second N-terminal Gc-site and the presence of the first of the two additional sites found more centrally in Gc of MPKV (**Fig 1**). In addition, signalase sites were more conserved with regard to GGV than MPKV (MPKV: Gn, VFS/A_17_P; NSm, INA/A_334_C; Gc, VKA/E_470_V). Overall, this characterizes K42904 as a divergent isolate of GGV, linking GGV to MPKV.

The increasing availability of sequence information for orthobunyaviruses indicates progressively more inconsistencies between the topologies of S, M and L phylogenetic trees. In the Mapputta clade, GGV was consistently closest to MPKV for all three segments, consistent with early serological data [5]; K13190 clustered with BUCV, and SW27571 was consistently closest to MURBV. In contrast, MAPV was for its L-segment closer to MPKV/GGV than to TRUV, but for its M-segment closer to TRUV than to MPKV/GGV. The S-segment phylogenetic analysis was less consistent with different histories inferred by different models, indicating that currently available sequences do not provide enough information to allow a statistically robust prediction (compare **Fig 2** and **S1 Fig**). In addition, whereas L-segment sequences of the Mapputta group formed a sister clade to all other orthobunyaviruses, their M-segment sequences branched within the orthobunyavirus clade, rooting Bunyamwera, Wyeomyia, California encephalitis, Bwamba and Wuhan louse fly clades. Again, analyses of the S-segment sequences were divergent, including low bootstrap support between 10 and 40% for deep nodes. Nevertheless, our findings for L-, and M-segments indicated differences in branching patterns compatible with early reassortment events and may indicate a divergent evolutionary history for these segments.

Our genetic analyses confirmed the absence of NSs coding sequence among all Mapputta group viruses as recently described for MAPV, MPKV, TRUV and GGV [10, 12, 14]. The NSs of bunyaviruses inhibits the induction of the cellular interferon response and deletion mutants show a reduced virulence [17, 26]. Similar to other NSs-lacking bunyaviruses of the Anopheles, Tete or Wyeomyia groups [19, 27], Mapputta group viruses may therefore be considered to have limited pathogenicity. However, bunyaviruses are also capable of using NSs-independent mechanisms to overcome the innate interferon response as indicated by Tacaiuma virus that suppresses interferon production despite the lack of an NSs [27], and known human pathogens such as Tataguine virus and Guama group viruses that do not encode NSs [12]. Shchentinin et al. showed that only 8 of the 15 sequenced serogroups encode this protein, suggesting that its presence or absence may not be a reliable predictor of pathogenicity [12].

Serosurveys indicate that both GGV and TRUV may infect humans. Seroprevalence rates were usually higher for GGV, reaching an average of 5–6% [7, 28]; however, TRUV was also linked to human infection although evidence for pathogenicity was less convincing [7]. Whereas cases of polyarthritic-like illness with a significant rise in GGV-specific IgM were recorded, cases with specific TRUV antibodies lacked in one instance detectable IgM and in another a concurrent infection with Ross River virus was suggested. To our knowledge, there is limited evidence for potential vertebrate hosts of GGV; with only one survey on the south coast of New South Wales reported, which found neutralizing antibodies most frequently in kangaroos and wallabies (33%), cattle (13%) or horses (13%)[29]. Serologic evidence of infection with TRUV has been reported in several species. Highest proportions of neutralizing antibodies were found in kangaroos (35–100%), wallabies (36–80%), and horses (46%) in sera collected in the 1950s and 1960s in Queensland [30]. The seroprevalence of TRUV neutralizing antibodies was also highest in kangaroos (71%) in a serosurvey conducted with samples collected at the south coast of New South Wales [29]. Similarly, in sera collected at several localities in southwest Western Australia neutralizing antibodies were highest in kangaroos (21%); antibodies were also detected in several other species, including feral pigs (4%), quokkas (5%), rabbits (up to 4%), horses (up to 3%) and foxes (10%) in individual localities [31]. In humans, seroprevalence rates of 0 to 14.0%, 1.4% and 0.3% have been reported in Queensland [30], New South Wales [7], and Western Australia [31], respectively. The predominant vector of TRUV, *Anopheles annulipes* s.l., displays opportunistic host-feeding behavior (reviewed in [32]), and this may be the reason that TRUV antibodies are found in a wide variety of vertebrates [31].

**Fig 3:**
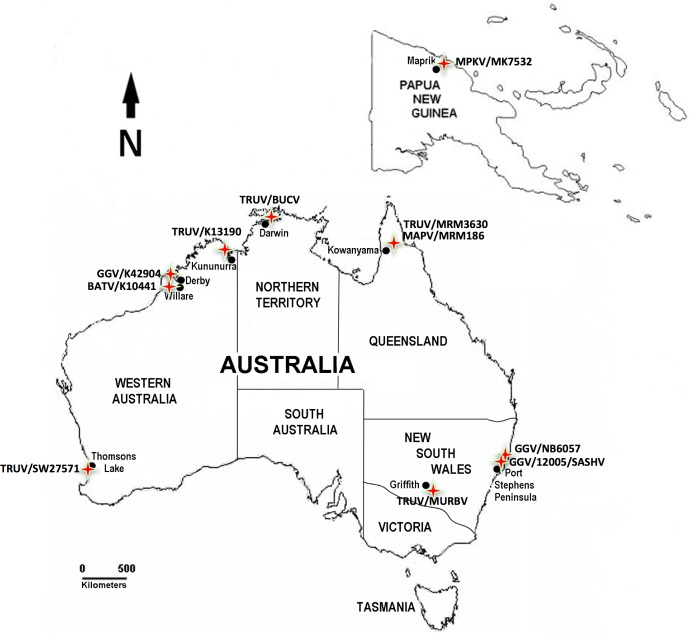
Geography of Australian Mapputta group viruses. MAPV, Mapputta virus; MPKV, Maprik virus; TRUV, Trubanaman virus; GGV, Gan Gan virus; MURBV, Murrumbidgee virus; SASHV, Salt Ash virus; BUCV, Buffalo Creek virus.

Geographically, TRUV appears to be circulating in *Anopheles* mosquitoes, primarily *An*. *annulipes*, throughout the Australian continent, including northern Queensland (Kowanyama), the Northern Territory (Darwin; BUCV), Western Australia (Kununurra in the Northwest, bordering the Northern Territory and Perth in the Southwest), as well as New South Wales (Griffith)(**Fig 3**). Prior to this study, GGV appeared to be focused in eastern Australia, mainly New South Wales (Port Stephens), involving various *Aedes* species, including the salt-marsh mosquito *Ae*. *vigilax*. The distribution of the sequence-confirmed isolates is largely in agreement with earlier reports of serologically characterized isolates (reviewed in [33]). However, K42904 obtained from a pool of *Ae*. *vigilax* collected in 2000 at Derby, Western Australia, extends the distribution of GGV-clade viruses into Western Australia.

Isolate K10441, obtained from *Cx*. *annulirostris* mosquitoes collected in 1993 at Willare, Western Australia, was identified as closely related to BATV, a virus classified in the species *Bunyamwera virus* of the genus *Orthobunyavirus* by the International Committee on the Taxonomy of Viruses (ICTV). This is the first detailed description on the occurrence of a BATV-clade virus from the Australian continent. The discovery of BATV dates back to 1955 when the prototype isolate MM2222 was obtained from *Cx*. *gelidus* mosquitoes collected in Kuala Lumpur, Malaysia (https://wwwn.cdc.gov/Arbocat/Default.aspx). With additional isolates from a variety of *Anopheles*, *Aedes* and *Culex* mosquitoes, as well as mammalian species in India (Chittoor virus), China (NM/12), Ukraine (Olkya virus), Europe (Calovo virus) and Uganda (UgMP6830)[34–40], BATV has to be regarded as one of the most widespread of the orthobunyaviruses [41, 42]. BATV is considered to persist in a mosquito-mammal cycle, including bovids, suids, cervids and leporids [34, 43–45]. While neutralizing antibodies have been found in human sera, mainly in Malaysia and Thailand, association with disease is ill-defined and infection may be limited to transient febrile illness with respiratory and/or gastrointestinal symptoms [46]. Given that the invasive mosquito species *Cx*. *gelidus* from which BATV was initially isolated in Malaysia was recently also reported in northern regions of Australia [47, 48], as well as other potential mosquito vectors of BATV, it is not surprising to encounter this virus in northern Australia. The occurrence in northern Australia may in fact represent another example of a virus potentially introduced into northern Australia from neighboring countries in Australasia or southeast Asia. Other such examples of suspected introductions into northern Australia include Japanese encephalitis virus [49, 50], or Bluetongue viruses and its vectors [51, 52].

In summary, we show the presence of Mapputta serogroup viruses throughout Australia, including Western Australia. The identification of K42904 from northern Western Australia adds a novel virus to the group that is genetically related to GGV and MPKV; thus linking eastern Australian GGV with Papua New Guinean MPKV. In addition, an orthobunyavirus from north-west Australia, closely related to BATV, was identified and comprehensively characterized for the first time. Given the serological cross-reactivity among the Mapputta group viruses (all belonging to the same serogroup), sero-diagnostic assignment of human and animal disease cases to distinct viruses should be considered tentative. Comprehensive sequence data will open the way to virus-specific nucleic acid tests for enhanced surveillance and clarifying the pathologic potential of each of these viruses in humans and animals.

## Materials and Methods

### Virus isolates and culture

Isolates K10441, K13190, and SW27571 were obtained from pools of mosquitoes collected during routine mosquito and arbovirus surveillance in Western Australia by The University of Western Australia Arbovirus Surveillance and Research Laboratory (UWA ASRL) using methods described previously [53–56]. Isolate 12005 was obtained from the UWA ASRL repository, and TRUV prototype MRM3630 and GGV prototype NB6057 were sourced from CSIRO Australian Animal Health Laboratory collection. Viruses were propagated in Vero cells for sequencing.

### Unbiased high-throughput sequencing (UHTS), reverse transcriptase—polymerase chain reaction (RT-PCR), and rapid amplification of cDNA ends (RACE)

Sequences were generated by applying a combination of consensus reverse transcriptase (RT)-polymerase chain reaction (PCR) and unbiased high-throughput sequencing (UHTS). Total RNA was extracted from culture supernatants using TRI Reagent (Molecular Research Center, Inc. Cincinnati, OH, USA) or RNeasy Plus Mini Kit (Qiagen, Hilden, Germany). Aliquots of total RNA extracts (0.5 μg) were treated with DNase I (Ambion, Austin, TX, USA or Promega, Madison, WI, USA) for reverse transcription (RT) by Superscript II (Invitrogen, Carlsbad, CA, USA) with random octamer primers linked to an arbitrary, defined 17-mer primer sequence. The cDNA was RNase H-treated for 454 sequencing or Klenow-treated for Ilumina sequencing and randomly amplified by PCR with AmpliTaq (Applied Biosystems, Foster City, CA, USA) and a primer mix including the octamer-linked 17-mer sequence primer and the defined 17-mer sequence primer in a 1:9 ratio [57]. Amplification products >70 bp were purified (MinElute, Qiagen) and ligated to linkers for sequencing on a GS-FLX Sequencer (454 Life Sciences, Branford, CT, USA)[58] or a MiSeq Sequencing system (Ilumina, San Diego, CA, USA). For the latter, Nextera XT DNA Sample Preparation Kit (Illumina) was used for library preparation and paired-end sequencing of 250bp fragments was performed with MiSeq reagent kit V2 (500 cycles; Ilumina). Sequencing data was analyzed using CLC Bio Genomics Workbench 6.5.0 (http://www.clcbio.com**).**

Direct PCR amplification was performed with conserved degenerate primer sets [19, 59, 60], or primer sets designed based on sequences obtained through UHTS. PCR reactions used routinely 1 μl random hexamer-primed cDNA (Superscript II; Invitrogen), primers at 0.2 mM concentration, and Platinum Taq DNA polymerase (Invitrogen). Amplification products were size-fractionated in 1.3% agarose gels, purified (QIAquick PCR purification kit; Qiagen), and either sequenced directly, yielding a majority sequence, or after cloning into pGEM-Teasy plasmid vector (Promega). Sequences were obtained for both strands by automated dideoxy-sequencing (Genewiz, South Plainfield, NJ, USA, or Applied Biosystems). Genomic termini were characterized by rapid amplification of cDNA ends using 5’-, or 3’-RACE (RACE kits; Invitrogen).

### Bioinformatics

Sequence reads were stripped of primer sequences and highly repetitive elements, quality filtered and then clustered and assembled into contiguous fragments (contigs) for comparison by the Basic Local Alignment Search Tool (blast [61]) to the Genbank database at nucleotide (nt; blastn) and deduced amino acid (aa; blastx) levels. Pairwise sequence identity percentages were calculated with the Needleman-Wunsch algorithm, applying an EBLOSUM62 substitution matrix (gap open/extension penalties of 10/0.5 for nt and aa alignments; EMBOSS [62]) and a Perl script to parse the results for all comparisons. Algorithms SignalP-NN/SignalP-HMM, NetNGlyc, and TMHMM (http://www.cbs.dtu.dk/services) were used for functional predictions. Phylogenetic analyses were performed by using the MEGA 6.0 software package [63]. Multiple sequence alignments were generated with the implemented Clustal algorithm. Phylogenetic histories were reconstructed based on translated amino acid sequence using the Neighbor-Joining method and applying the Poisson substitution model. Bootstrap values were calculated based on 1000 pseudoreplicates. In addition, an analysis using the Maximum-Likelihood method was performed using 50 pseudoreplicates and the best predicted substitution model for the alignment.

## Supporting Information

S1 Fig Phylogenetic relationship of Australian Mapputta group and K10441 isolates to other selected orthobunyaviruses. Deduced amino acid sequences of the S- (N ORF; panel **A**), M- (Gn, NSm,Gc polyprotein ORF; panel **B**), and L-segment (RdRp-ORF; panel **C**) were aligned and trees reconstructed with the Maximum Likelihood method applying the best predicted substitution model using MEGA 6 software. Bootstrap values are indicated at the respective nodes, scale bars indicate the number of substitutions per site, and GenBank accession number and isolate name (where known) are given next to the virus name. MAPV, Mapputta virus; MPKV, Maprik virus; TRUV, Trubanaman virus; GGV, Gan Gan virus.
(TIFF)

S1 Table Nucleotide and amino acid sequence identities. Percent nucleotide (NT) and amino acid (AA) sequence identity among Mapputta group viruses (**a**), and among isolate K10441 and selected other orthobunyaviruses (**b**) were calculated.
(XLSX)
