# Supplementary figures and images for: Analysis of Arbovirus Isolates from Australia Identifies Novel Bunyaviruses Including a Mapputta Group Virus from Western Australia That Links Gan Gan and Maprik Viruses
Source: PLoS One. 2016 Oct 20;11(10):e0164868. doi: 10.1371/journal.pone.0164868 (PMC5072647; doi:10.1371/journal.pone.0164868)

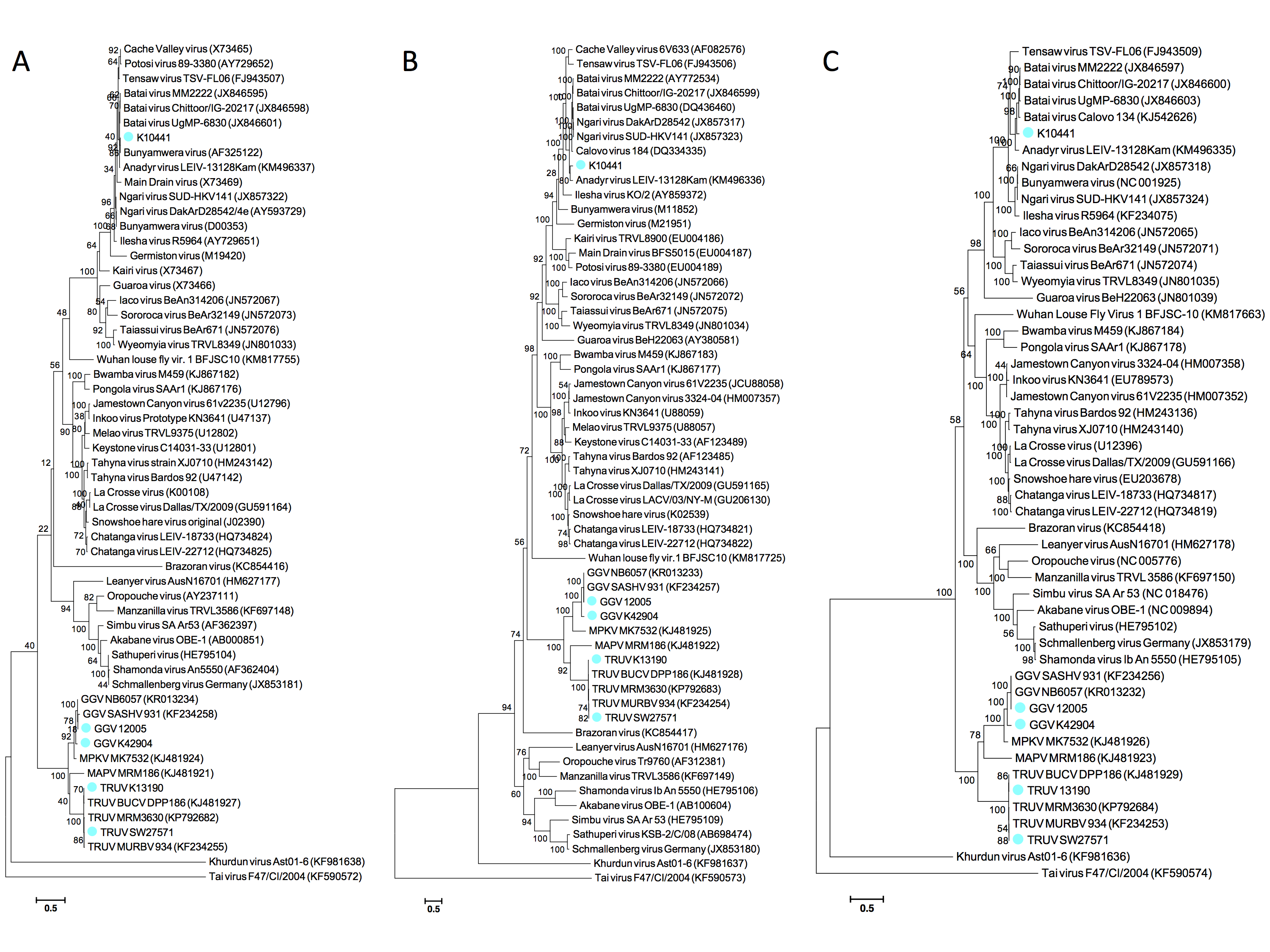

Supplement: S1 Fig — Deduced amino acid sequences of the S- (N ORF; panel A), M- (Gn, NSm,Gc polyprotein ORF; panel B), and L-segment (RdRp-ORF; panel C) were aligned and trees reconstructed with the Maximum Likelihood method applying the best predicted substitution model using MEGA 6 software. Bootstrap values are indicated at the respective nodes, scale bars indicate the number of substitutions per site, and GenBank accession number and isolate name (where known) are given next to the virus name. MAPV, Mapputta virus; MPKV, Maprik virus; TRUV, Trubanaman virus; GGV, Gan Gan virus. (TIFF) [file pone.0164868.s001.tiff]
